# Self-efficacy, Stress and Well-being in the transition to Higher Education

**DOI:** 10.1192/j.eurpsy.2023.1024

**Published:** 2023-07-19

**Authors:** J. Cruz, R. Lopes

**Affiliations:** 1 Unidade Intermédia Médico-Cirúrgica/Serviço de Cuidados Intensivos 1, Centro Hospitalar Universitário do Porto – Hospital de Santo António, Porto; 2 UCP Enfermagem de Saúde Mental e Psiquiátrica, Escola Superior de Enfermagem de Coimbra, Coimbra, Portugal

## Abstract

**Introduction:**

The transition from secondary education to higher education (HE) marks the beginning of a new stage in the individual journey of students, which is assumed to be one of the best and most remarkable periods of life.

University students constitute a risk group in which situations that generate stress are abundant and potentially disturbing, which can condition their self-efficacy and perception of well-being.

**Objectives:**

Describe correlations between sociodemographic variables and self-efficacy, perceived stress and psychological well-being;

Understand the correlation between the various variables under study in newly admitted students in a HE establishment;

Raising awareness of the importance of the Specialist Nurse in Mental Health and Psychiatric Nursing in the transition process, promotion of mental health and prevention of mental illness.

**Methods:**

Descriptive and correlational study with a non-probabilistic sample of students in the 1^st^ year of the nursing degree at a Portuguese nursing school.

Data collection took place in the 1^st^ semester of the 2019/2020 school year, after a favourable opinion from the Ethics Committee and authorization from the HE institution’s governing bodies. The following measurement instruments were used: Sociodemographic/Academic Questionnaire, General Self-Efficacy Scale, Perceived Stress Scale and Psychological Well-Being Manifestation Scale.

**Results:**

There are statistically significant differences between global self-efficacy and the variables that measure who students live with during the school year, whether entering HE implies leaving home, participation in extracurricular activities, professional activity, level of adaptation to the institution of education and level of schooling satisfaction with the course. There are statistically significant differences between perceived stress and the variables gender, studying away from home, who they live with during school term, participation in extracurricular activities, level of adaptation to the institution, degree of satisfaction with the course and need for psychological support.

There were statistically significant differences between psychological well-being and gender and variables measuring necessity studying away from home, who they live with during the school term, participation in extracurricular activities, economic situation, level of adaptation to the institution and degree of satisfaction with the course.

There was a negative correlation between general self-efficacy and perceived stress (moderate) and between perceived stress and psychological well-being (strong) and a moderate positive correlation between general self-efficacy and psychological well-being of HE students.

**Conclusions:**

It is concluded that the transition environment for HE is complex and impactful for students, so it is essential to develop facilitating strategies in order to reduce the impact of stress-inducing factors and emotional exhaustion in this population.

**Disclosure of Interest:**

None Declared

